# A new giraffe ossicone from Wolf Camp, Tunggur Formation, Inner Mongolia suggests a new genus in Bohlinini (Artiodactyla, Giraffidae)

**DOI:** 10.1371/journal.pone.0328405

**Published:** 2025-08-05

**Authors:** Xiaoming Wang, Nikos Solounias, Su-kuan Hou, Lu Li, Yukimitsu Tomida

**Affiliations:** 1 Department of Biological Sciences, University of Southern California, Los Angeles, California, United States of America; 2 Department of Vertebrate Paleontology, Natural History Museum of Los Angeles County, Los Angeles, California, United States of America; 3 Key Laboratory of Vertebrate Evolution and Human Origins, Institute of Vertebrate Paleontology and Paleoanthropology, Chinese Academy of Sciences, Beijing, China; 4 Department of Paleontology, American Museum of Natural History, New York, New York, United States of America; 5 Department of Anatomy, New York Institute of Technology, College of Osteopathic Medicine, Old Westbury, New York, United States of America; 6 College of Earth and Planetary Sciences, University of Chinese Academy of Sciences, Beijing, China; 7 Department of Geology and Paleontology, National Museum of Nature and Science, Tsukuba, Ibaraki, Japan; CONICET: Consejo Nacional de Investigaciones Cientificas y Tecnicas, ARGENTINA

## Abstract

The classic Middle Miocene Wolf Camp locality discovered in 1930 by the Third Asiatic Expeditions of the American Museum of Natural History has been long known to produce an extinct giraffe, *Palaeotragus tungurensis* Colbert, 1936. Its dental and limb morphology offers tantalizing clues to a close relationship to the living giraffe, *Giraffa*. Its ossicone, a key part of the giraffe anatomy, is unknown since its initial description. Our discovery, in 2011, of an almost perfectly preserved ossicone from Wolf Camp thus fills this void and is described herein. Novel morphology of the ossicone, unlike any known so far, warrants a new generic name, *Qilin*, and *Q. tungurensis* adds important evidence that this Middle Miocene record from Inner Mongolia represents a key taxon in the evolution of the subfamily Giraffinae. Ossicone morphology is fundamentally similar to that of living *Giraffa* as well as members of Bohlinini. Dentally, *Q. tungurensis* also strongly supports its membership within Bohlinini with a unique shared derived character of P2-3 para- and metastyles bending inward to mesostyle. *Q. tungurensis* possesses a slender limb and a modestly deep trough on the posterior surface of the metacarpals that also suggest membership in Bohlinini. Combined with knowledge about its dental and limb morphology, the new Wolf Camp ossicone indicates an important stage of giraffine evolution, and contributes to a better understanding of its chronology and zoogeography.

## Introduction

In 1936, Edwin Colbert named a new species of an extinct giraffe, *Palaeotragus tungurensis*, based on a partial skull plus several maxilla and lower jaws from the Middle Miocene Tunggur Formation of Inner Mongolia and collected during the Third Asiatic Expeditions of the American Museum of Natural History [1]. The type skull belongs to a female without an ossicone, but several authors have placed the Tunggur form at the base of the modern giraffe lineage (Giraffinae) based on dental and postcranial similarities [2–4]. Unfortunately, information on the ossicone for the Tunggur fossil material is still lacking more than 90 years after initial discovery.

In our 2011 field season, a nearly complete left ossicone was discovered in the Wolf Camp section of the Tunggur Tableland. Not only is this the most precisely documented occurrences of giraffid material in the Tunggur Formation (previously published specimens were all recorded from the “Wolf Camp quarry” without details about their biostratigraphy within the section), but more importantly, the novel morphology of the new ossicone makes it possible to re-evaluate its systematic relationship. New information about the ossicone morphology seems consistent with a long-held view that the Inner Mongolian form is likely an early member of the tribe Bohlinini, which in turn may be the sister of living *Giraffa* [3].

Additionally, the Middle Miocene age for the Tunggur Formation is also unique among Chinese fossil giraffe records. The Inner Mongolian record is the earliest giraffid in China, well before the occurrences of most giraffids in the late Miocene of North China [2]. This makes the Tunggur forms valuable for a better sense of early records in China, despite the fact that Early and Middle Miocene Bugti and Siwaliks strata in Pakistan appear to have the highest diversity of giraffoids, such as *Progiraffa*, *Ua*, *Giraffokeryx*, *Orea* and others [5,6].

## Materials and methods

### Institutional abbreviations

**AMNH**, American Museum of Natural History, New York, New York; **IVPP**, Institute of Vertebrate Paleontology and Paleoanthropology, Chinese Academy of Sciences, Beijing; **MEUU**, Museum of Evolution at Uppsala University, Uppsala; **NRM**, Naturhistoriska Riksmuseet, Stockholm; **PRCI**, Paleontological Research Center of Iran, Maragheh; **SNSB-BSPG**, Staatliche Naturwissenschaftliche Sammlungen Bayerns—Bayerische Staatssammlung für Paläontologie und Geologie, Munich.

### 3D models by laser scans

The new ossicone was scanned using a NextEngine scanner (model 2020i) in combination with ScanStudio software (version 2.0.2). The specimen was scanned in the highest resolution in the “Macro” setting, which has a 0.005” accuracy (~40,000–160,000 points/square inch or about 6,200–24,800 points/square cm). Scans were saved in the PLY format that preserves texture information. Size scale was captured by build-in calibrations of the NextEngine scanner.

Virtual cross sections of the ossicone are derived from above 3D model using MeshLab’s (v2020.07; https://www.meshlab.net/) build-in function of “Compute Planar Section”. The cross-section outlines were then traced in Adobe Illustrator.

### microCT scan

The new ossicone was also micro-CT scanned using a Bruker SkyScan 1273 model by Micro Photonic with a vortex size of 50.401162 um (X/Y Ratio = 1.0). We used the factory software CTvox (version 3.3.1 64-bit) by Bruker microCT for initial image adjustments, manipulation, and virtual sections. Composite 2D images from screen saver were processed by PhotoShop.

### MorphoSource repository

MorphoSource is a repository platform (www.morphosource.org) that holds digital data of biological specimens and cultural heritage objects contributed by museums, researchers, and scholars. We have uploaded 3D model and original CT scan files pertaining to *Qilin tungurensis* to this site (https://www.morphosource.org/concern/media/000711877?locale=en) to make them broadly available.

## Systematic paleontology

Order Artiodactyla Owen, 1848 [7]

Suborder Ruminantia Scopoli, 1777 [8]

Infraorder Pecora Linnaeus, 1758 [9]

Superfamily Giraffoidea Gray, 1821 [10]

Family Giraffidae Gray, 1821 [10]

Subfamily Giraffinae Gray, 1821 [10]

**Included tribes**: Giraffini Gray, 1821 (living giraffe and its relatives) and extinct Bohlinini Solounias, 2007.

**Remarks**: Bohlin [11] placed his newly named *Honanotherium* plus *Bohlinia* (then known as *Orasius*) in the subfamily Giraffinae, which includes the living giraffes. Bohlin’s taxonomic framework proves to be enduring since then, as it was variously followed by Matthew [12], Colbert [13], Hamilton [4], Solounias [3], Ríos et al. [14], and Parizad et al. [15], but see Geraads [16] and Ríos et al. [17] for alternative arrangements. As the associated limbs and necks become better known, the evolution of lengthening necks and limbs is also consistent with this framework [4]. Solounias [3], however, pointed out that in the development of a shallow trough on the posterior face of metacarpals, living giraffes lacks this structure in contrast to those of his newly named Bohlininae (including *Bohlinia* and *Honanotherium*) with deep troughs (see more discussions under Tribe Bohlinini). He therefore proposed two alternative scenarios in *Giraffa* relationships to account for the metapodial characters, i.e., *Giraffa* either evolved from within Bohlininae or *Giraffa* was close to *Palaeotragus rouenii*.

### Tribe Bohlinini Solounias, 2007 new rank

**Included genera**: *Bohlinia* Matthew, 1929; *Honanotherium* Bohlin, 1926; and *Qilin* new genus.

**Remarks**: Based on a “working cladogram,” Solounias [3] proposed a new subfamily Bohlininae (roughly equivalent to Bohlinini here) that includes *Injanatherium*, *Honanotherium*, *Decennatherium*, “*Palaeotragus*” *tungurensis*, *Bohlinia*, and *Birgerbohlinia*. Solounias’ original concept places Bohlininae near the base of his Giraffoidea tree. His new Bohlininae clade was an attempt to account for the metapodial morphology with their long and slender metacarpals and a deep trough posteriorly at the proximal cross sections [3:fig 21.7D]. He further hypothesized that the characteristic deep troughs and presumably well-developed palmar interossei to adduct the digits are probably related to habitats of soft substrates such as muddy lake margins or sandy areas. In addition to Bohlininae’s shared metacarpal morphology [node 8 of 3], successive nodes (8–12) of derived characters in Solounias’ Bohlininae clade include large size and one pair of ossicones (node 10; *Honanotherium*, “*Palaeotragus*” *tungurensis*, *Bohlinia*, and *Birgerbohlinia*), upper premolars with inward-curving styles in *Bohlinia* but not in *Honanotherium* and premolars round in occlusal view (node 11; “*Palaeotragus*” *tungurensis*, *Bohlinia*, and *Birgerbohlinia*), and large-sized species (node 12). Danowitz et al. [18] added that in addition to long and slender metapodials with a deep trough, Bohlininae is also characterized by the lack of cranial sinuses, but this latter feature seems likely a primitive character.

Although knowledge of the neck vertebrae in *Bohlinia* is still incomplete, known cervicals (two specimens of C2 from Pikermi in Stockholm collection) suggest a strikingly lengthened neck, on par to species of *Giraffa* [18]. Thus a potential sister relationship of Bohlinini and Giraffini, or even *Giraffa* being derived from within Bohlinini [3], is a possibility. However, the C7 in *Bohlinia* from Pikermi shows a simple, typical C7 unlike that of Giraffini.

Parizad et al. [15] described skulls of *Bohlinia attica* from the late Miocene Maragheh fauna of Iran. Ossicones of the Maragheh skulls (PRCI/M352 and M353) share many similarities to our new Wolf Camp ossicone. If we equate the anterior “flange” on the Tunggur specimen with the ridge of known *Bohlinia* ossicones, then a case can be made that our new Tunggur ossicone belongs to Bohlinini. On the other hand, if the flange is interpreted to be derived from a separate ossification center, then a Bohlinini affinity can still be postulated based on dental characters (inwardly bending parastyle and metastyle in upper premolars), plus the blunt tip of ossicone and terminal expansion at the tip with a constriction below the tip.

Another important genus frequently invoked in discussions about the content of Bohlinini/Bohlininae is *Honanotherium*, which has a convoluted history of nomenclature. Based on fossils purchased from Chinese drug stores by K. A. Haberer, Schlosser [19:pl. IX] illustrated various dental materials and referred them to *Camelopardalis sivalensis* Falconer and Cautley, 1843. Without designating a holotype, Pilgrim [20] proposed a new species, *Giraffa schlosseri*, based on his observation that Schlosser’s Chinese dental materials have relatively small premolars compared to molars. Bohlin [11] established a new genus *Honanotherium* for Pilgrim’s *G. schlosseri*, but his concept of this genus was largely based on the newly acquired Lagrelius Collection housed at Uppsala University, i.e., the original drug store dental materials (in SNSB-BSPG, Munich), being less diagnostic, were no longer the main basis of *H. schlosseri*. The Lagrelius Collection for Bohlin’s *H. schlosseri*, however, were further complicated by the fact that they came from ten localities (11, 12, 13, 27, 29, 35, 58, 59, Sung-Tsun, and Yang-Kou) mostly in Xin’an County of Henan Province, eight localities in Baode (localities 44 and 49) and Yushe (localities 70, 73, 77, 78) basins in Shanxi Province, and one locality in Fugu area (locality 51) of Shaanxi Province. Bohlin’s main concept of his *Honanotherium* was influenced by two well-preserved skulls from Henan Province, hence his generic name. As Pilgrim, Bohlin did not assign a type specimen either. Solounias and Danowitz’s [21] attempted to fix the type by selecting MEUU M 3886 as the lectotype. However, their lectotype designation was not based on the original (mostly dental) syntype series purchased from the drug store, as was stipulated for lectotypes to be a valid designation by the International Commission on Zoological Nomenclature [22:article 74.2]. Questions, therefore, remain whether or not Schlosser’s drug store materials include diagnostic teeth to be selected as a lectotype and if not, the nominal species, *Honanotherium schlosseri*, may possibly be designated a *nomen vanum* [23]. In this regard, Bohlin [11:110] remarked that Schlosser’s M3 length and width ratio for *H. schlosseri* is consistent with that of the modern giraffe but it would seem prudent to wait for further evaluation.

In this paper, we continue to use *Honanotherium schlosseri* in its conventional understanding despite the above nomenclatural problems. Solounias and Danowitz [21] discussed similarities between *Honanotherium* and *Bohlinia*, including the ossicone morphology, skull structure and the depth of the metapodial trough. The limbs of *Honanotherium*, however, are shorter than those of *Bohlinia*. In overall skull proportions, *Bohlinia* is also more similar to living *Giraffa*.

Membership of *Birgerbohlinia schaubi* within Bohlinini, as proposed by Solounias [3], is also in question. Although this Spanish Turolian taxon does appear to have inwardly curving styles on its P3 [24], it has two pairs of ossicones [25] that stand out from many other giraffines. In most cladistic analysis, *Birgerbohlinia* are often placed near *Samotherium* and *Sivatherium* [14,17] or *Giraffokeryx* [4]. Similarly, phylogenetic positions of other genera included in Solounias’ Bohlininae, such as *Injanatherium* and *Decennatherium*, are controversial, with the former hypothesized as a sister of *Giraffokeryx* and the latter as a basal sivatheriine [14,17].

### *Qilin* new genus

**Type species**: *Palaeotragus tungurensis* Colbert, 1936.

**Included species**: Type species and an undescribed species from the Siwaliks of Pakistan [5].

**Diagnosis**: Thin and slender metacarpal with a deep posterior groove; premolar parastyles and metastyles prominent and bending inward; ossicone with an upright shaft, curving inward (medially), expanded tip and a slight constriction below the tip, and a greatly expanded posterior flange with surface bumps.

**Etymology**: *Qilin*, Chinese Pinyin spelling for 麒麟, a mythical creature in ancient Chinese texts with a deer body, cow tail, and single (or paired) antler with fleshy tip; Qilin was invoked as an approximate transliteration for Somali word “geri” for giraffe when the Ming dynasty maritime expedition to Africa brought two giraffes to Beijing in 1414 A.D. 麒麟 as giraffe is still used in Korean Hanja and Japanese Kanji writing systems, as well as in early Chinese scientific literatures as a common name for “giraffe deer family” (麒麟鹿科), such as in the Chinese abstract of Bohlin [11].

**Remarks**: Colbert [1] noted the inwardly turning styles in upper premolars that are unique in his newly established *Palaeotragus tungurensis* as well as other dental similarities to several giraffids, such as *Giraffokeryx* and *Samotherium*. He, however, saw no need of naming a new genus because he regarded the Tunggur form as most closely related to *P. rouenii* and other *Palaeotragus* spp. Hamilton [4] was apparently first to hypothesize that the Inner Mongolian “*Palaeotragus*” *tungurensis* may be closely related to the Giraffinae. As such, he placed it under quotation marks (shown only in his abstract, cladogram, and list of classification; in the rest of his text, he did not have quotation marks), which may also include *P. primaevus* from Kenya, a recognition that the Tunggur form needs a genus name of its own. Solounias [3:259] followed Hamilton’s treatment of “*Palaeotragus*” *tungurensis* and remarked that it required a “new systematic name,” as also implied in Solounias and Danowitz [5] in their treatments of undescribed taxa from the Siwaliks of Pakistan. Hou [2] also continued the above practices by Hamilton and Solounias in highlighting this taxonomic issue. With our new knowledge of the ossicone morphology presented here, we are now in a position to erect a new genus to mark its unique taxonomic position.

### *Qilin tungurensis* (Colbert, 1936), new combination

*Palaeotragus tungurensis* Colbert, 1936:1, in part; Churcher, 1970:86.

“*Palaeotragus*” *tungurensis* Colbert: Hamilton, 1978:166, fig 9; Solounias, 2007:260, fig 21.1; Parizad et al., 2019:32; Hou, 2023:213.

**Fig 1:**
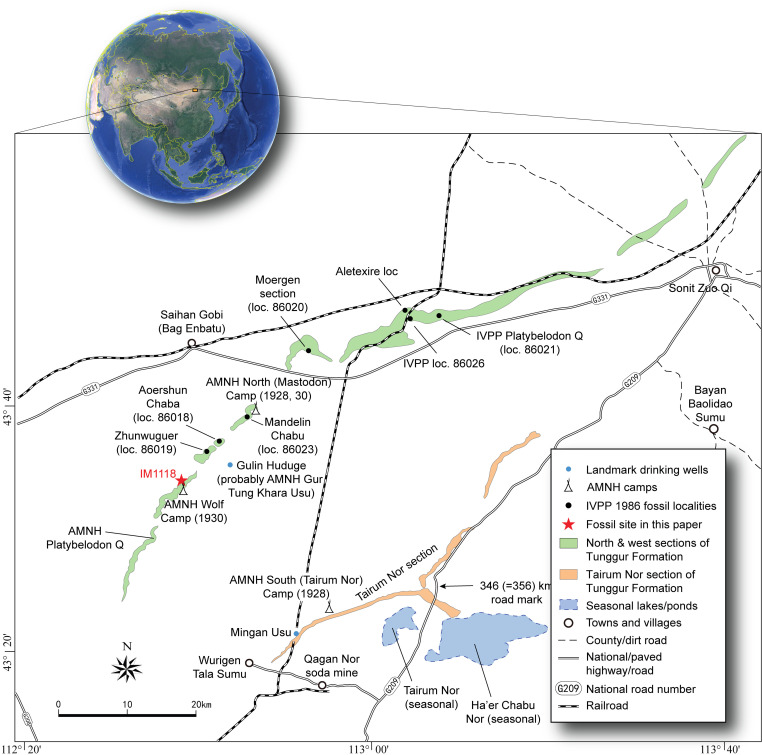
Distribution of Tunggur Formation exposures and relationships of AMNH 1928-1930 and IVPP 1986 localities, modified from Wang et al. [28:fig 1]. AMNH localities were based on archival studies by Wang et al. [27] and IVPP 1986 localities were based on plots in IVPP topographic maps and Qiu Z.-x. et al. [29].

**Fig 2:**
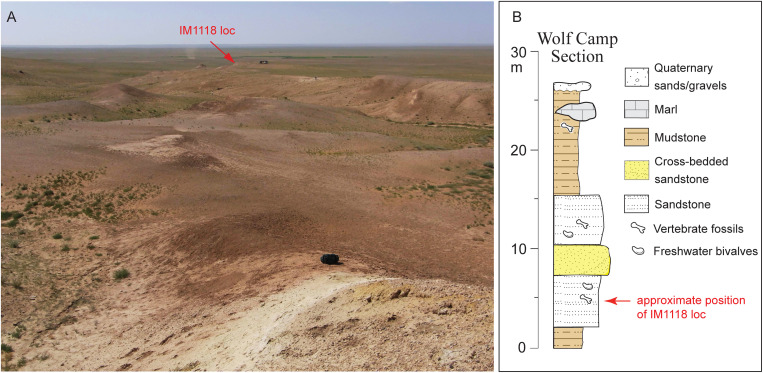
(A) photo of Wolf Camp exposures and approximate location of IVPP IM1118 locality (red arrow), field vehicles to the right of red arrow for scale, photo taken by Yukimitsu Tomida on August 10, 2011; B, measured section of Wolf Camp exposures, modified from Wang et al. [27:fig 8].

**Fig 3:**
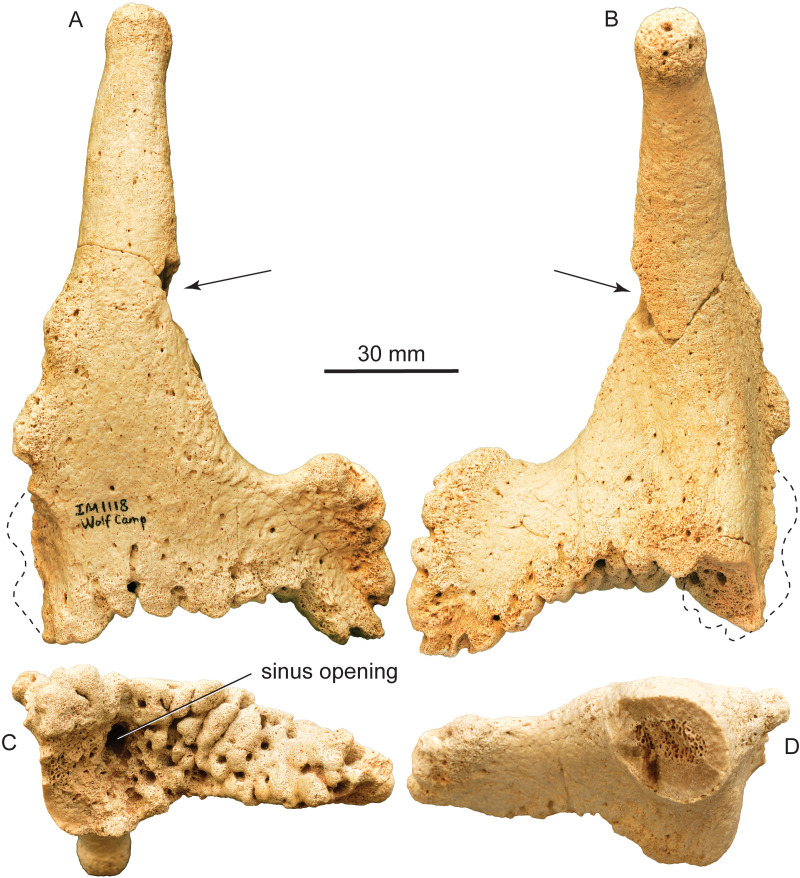
*Qilin tungurensis* [1], IVPP V 33851, right juvenile male ossicone from IVPP locality IM1118, lower part of Wolf Camp section. (A) right lateral view; (B) medial view, black arrows indicating natural break before the ossicones were glued back together (see D); (C) ventral (proximal) view; and (D) dorsal view before the upper shaft was glued (black arrows in A and B showing location of the break), showing oval cross-section at mid shaft.

**Holotype**: AMNH 26582, a partial skull with right P2-M3 [1:figs 1, 3, 2:fig 131] (Figs 6, 7A).

**Fig 4:**
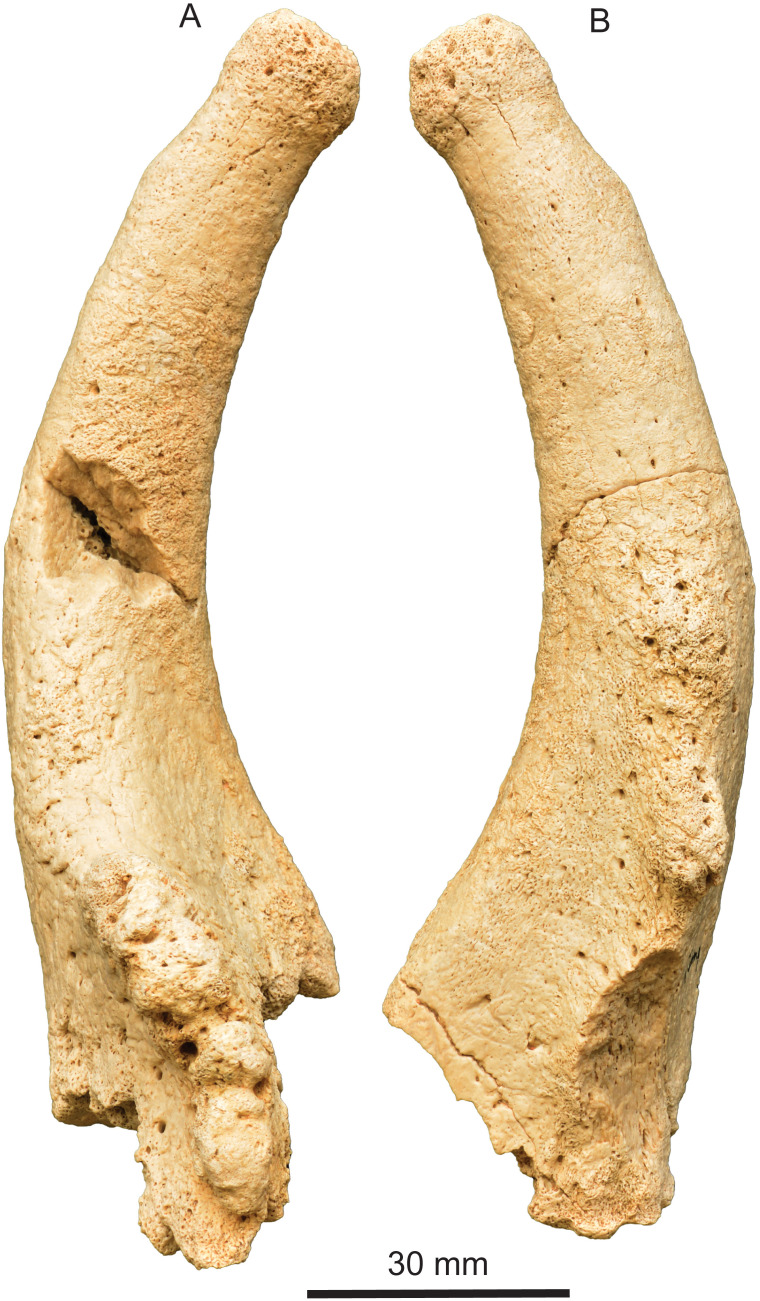
*Qilin tungurensis* [1], IVPP V 33851, right ossicone from IVPP locality IM1118, lower part of Wolf Camp section. (A) anterior view; and (B) posterior view.

**Fig 5:**
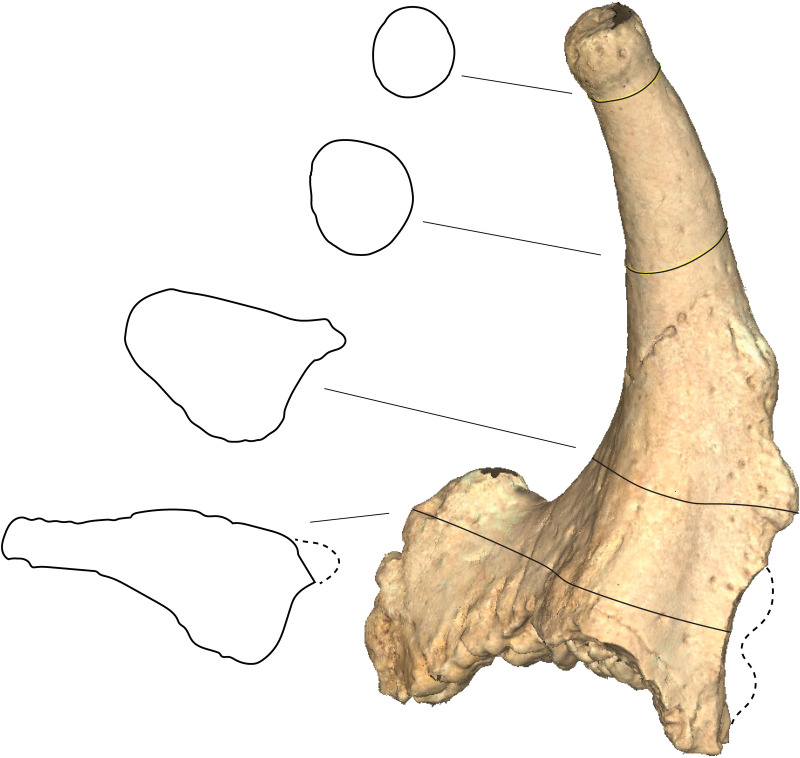
Successive cross sections of ossicone for *Qilin tungurensis* [1], IVPP V 33851. Dash lines are estimated missing overgrowth bumps along the posterior ridge. Virtual cross sections (left) of the ossicone are derived from a 3D laser scan model (right) using MeshLab’s (v2020.07; https://www.meshlab.net/) build-in function of “Compute Planar Section”. The cross-section outlines were then traced in Adobe Illustrator. The 3D model file can be downloaded from MorphoSource.org (see Materials and Methods).

**Referred specimens**: IVPP V 33851, nearly complete right ossicone (Figs 3–5), likely a juvenile male, from IVPP IM1118 locality (N43°33’08.5“ E112°39’05.8”) in Wolf Camp area, about 600 m northwest of the AMNH Wolf Camp (Figs 1 and 2), collected and recorded by Yukimitsu Tomida on August 10, 2011.

Additional cranial and dental materials from the Wolf Camp area collected during the AMNH Central Asian Expeditions in 1930 include [following 2]: AMNH 26583, left and right dentary with complete dentition [2:fig 132]; AMNH 26584, right palate with P3-M3; AMNH 26585, palate and left dentary with worn teeth; AMNH 26586, palate with left and right P2-M3 and a left dentary with p2-p4, paratype (Fig 8B); AMNH 26587, left palate with DP2–4 and M1-2; AMNH 26588, broken palate with left and right DP2–4 and M1; AMNH 26590, right dentary with dp2–4 and m1; and AMNH 26591, right dentary with p4-m3.

**Fig 6:**
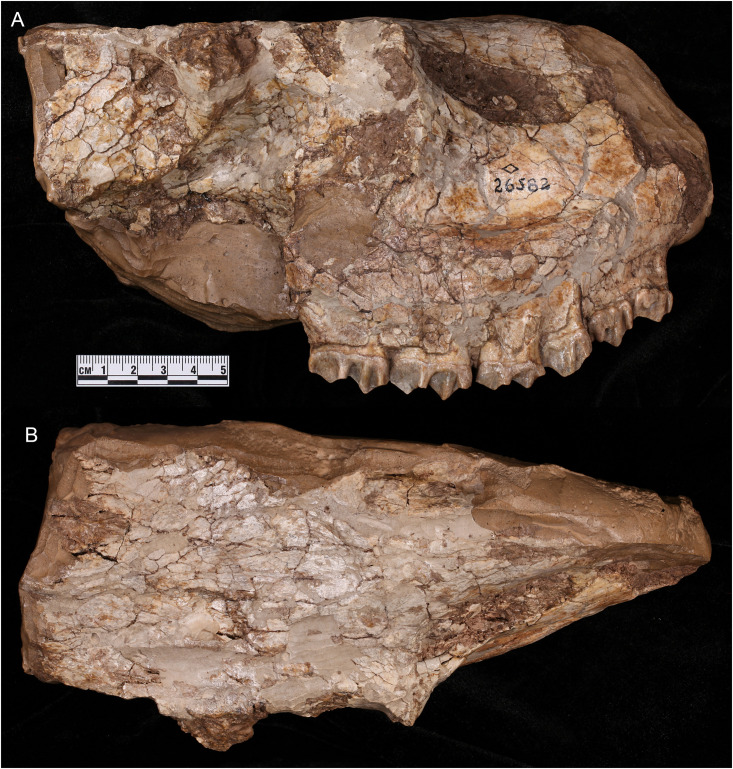
Partial skull of *Qilin tungurensis* [1], AMNH 26582, holotype from Wolf Camp, probably a female, showing no sign of attachment area for the ossicone. (A) lateral, and (B) dorsal views. Photos by Wei Gao.

**Fig 7:**
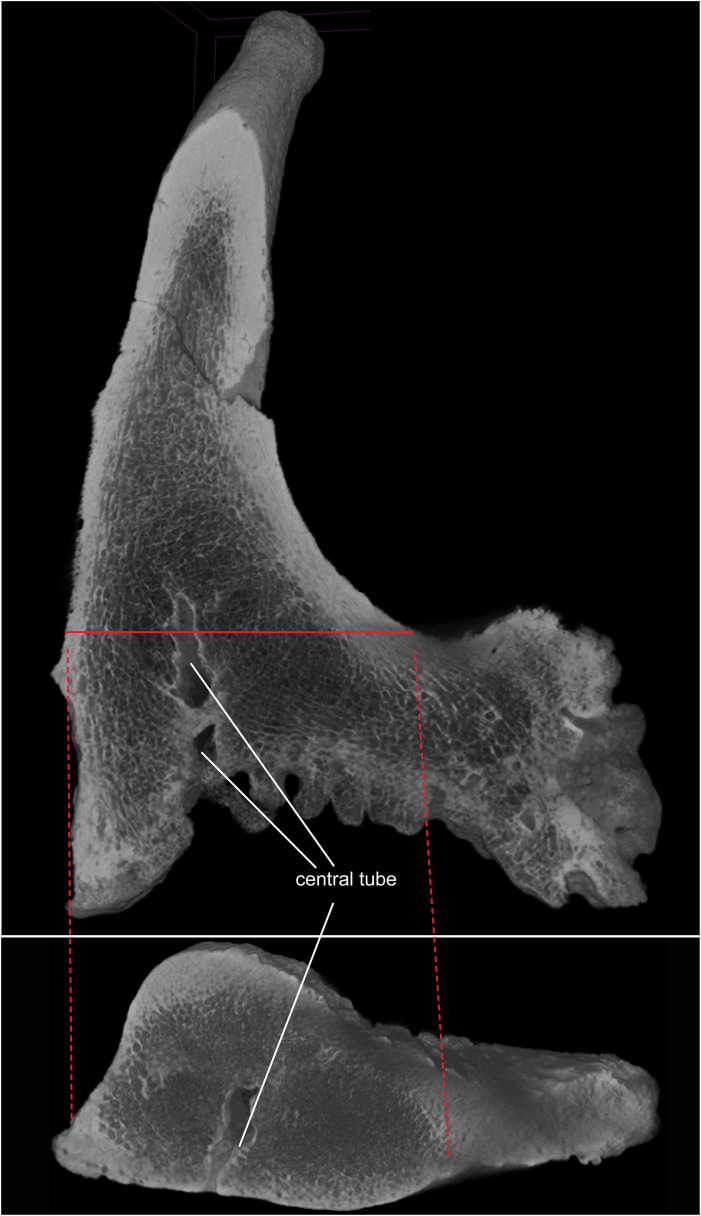
Virtual sagittal (upper) and cross (lower) sections of the ossicone of *Qilin tungurensis* (IVPP V 33851) from a microCT scan showing internal structures (see Material and methods section for download of original CT data). Note that in the cross-sectional view, the central tube penetrates the ossicone wall and emerge to external surface. Solid red line indicates approximate location of cross section.

Although not mentioned in Colbert’s original description [1], Solounias [3:fig 21.7D] illustrated two partial metapodials, presumably from the Wolf Camp, that may also belong to this species: AMNH 92306, distal metatarsal; and AMNH uncatalogued (field number 808), proximal metacarpal.

Hou [2] listed additional referred specimens including two incomplete lower jaws with m2-3 and p3-m3 (IVPP V 3209 and 3209.1) from the Middle Miocene Lengshuigou Formation in Lintong, Shaanxi Province, which were previously published as *Palaeotragus* sp. [26].

**Type locality and chronology**: Colbert [1] stated that all of the materials of his *Palaeotragus tungurensis* came from “Wolf Camp Quarry.” However, as far as we are aware, there isn’t a single quarry at the AMNH Wolf Camp area, in contrast to AMNH Platybelodon Quarry that is a true quarry producing multiple individuals of the shovel-tusked proboscidean *Platybelodon grangeri*. Instead, the AMNH Wolf Camp locality represents a large area of exposures, up to one km wide, with a total thickness of the local section >25 m [27] (Figs 1 and 2). From our field experience during the past 30 years, vertebrate fossils are found throughout the section, with the possible exception of the top marly layers. Fossils are more abundant in the lower sandstones, although in recent years, sand dune encroachments tended to fill up the lower sections of the wash, reducing fossiliferous exposures.

The new ossicone, IVPP V 33851, was from IVPP IM1118 locality (N43°33’08.5“ E112°39’05.8”), northwest of the AMNH 1930 Wolf Camp, at lower part of the section in the Wolf Camp area (Fig 2; position of the AMNH 1930 camp based on archive photos, See Wang et al., 2003). Despite it being the richest fossil site in the Tunggur Formation with highest diversity [27,29], the Wolf Camp section never has a palaeomagnetic study. Using the cross-bedded channel sandstone with abundant fossil mollusks as marker beds, Wang et al. [27] correlated the Wolf Camp section with the upper part of the Moergen section. A paleomagnetic study was published on the Moergen section [27], and Z.-d. Qiu et al. [30] correlated the magnetic section to C5r.3r through part of C5Ar.2r, which has a GTS2020 calibrated age of 11.657–12.829 Ma [31]. If such a correlation is correct, the Wolf Camp section should fall within the range of ~11.7–12.8 Ma in the late Middle Miocene, consistent with a Tunggurian Chinese Land Mammal Age [32].

**Diagnosis**: Same as for genus.

**Description and comparison of ossicone**: Here we mainly treat the newly discovered ossicone from Wolf Camp (IVPP V 33851) (Figs 3–5; Table 1). For cranial and dental morphology of *Qilin tungurensis*, see Colbert [1] and Hou [2] for more detailed descriptions of this species. See also Solounias [3:fig 21.7D] for its metacarpal morphology. Throughout the text, our usage of two ossicones refers to a single pair ossicones on each side of the skull, i.e., a single pair of ossicones are present in contrast to four ossicones, i.e., two pairs (four) ossicones.

**Table 1:** Measurements of ossicones (in mm). See Parizad et al. [15:table 1] for definitions and Kostopoulos et al. [33:fig 2] for illustrations of ossicone measurements.

	*Qilin tungurensis*	*Bohlinia attica*					
	Tunggur (this paper)	Maragheh [15]		Nikiti-1 [33:table 10]			Pikermi [11]
	IVPP V 33851	PRCI/M353	PRCI/M352	NKT-52	NKT-145	NKT-147	NRM M4420
Total length (height) along anterior margin	154.5	202	170+		175+	190+	~225
Transverse diameter at the base of the ossicones	41.5	79	63.8	73.0	68.8	72.0	51
Antero-posterior diameter at the base of the ossicones	81.6	98.5	71	135.0	135.5	152.0	62
Transverse diameter of the ossicones at 10 cm from the base	20.4	52.2	36.9	38.0	48.0	53.0	
Antero-posterior diameter of the ossicone at 10 cm from the base	19.6	40.1	37.2	44.0	54.0	54.0	

IVPP V 33851 is a nearly perfectly preserved ossicone, missing only some small bumps along the posterior ridge at the base of the ossicone plus a small corner piece at the basal attachment area. These minor imperfections do not prevent a good understanding of the entire ossicone. A major shortcoming of IVPP V 33851, however, lies in its detachment from its skull, thus offering few clues about its orientation.

The main shaft of the ossicone is perfectly upright in medial or lateral views, i.e., nearly perpendicular to the basal attachment surface, in contrast to the majority of giraffid ossicones that either have a recumbent orientation or a posterior curvature of the main shaft, or both [14:figs 33, 34]. Such a posteriorly leaning/curving ossicone in most giraffids can thus help to identify ossicone positions. Unfortunately, the upright orientation of IVPP V 33851 offers no such clue and whether or not it is a left or right ossicone remains to be settled pending better preserved cranial materials.

In his original description, Colbert [1] was mostly concerned with dental morphology and did not describe the cranial morphology of his *Qilin tungurensis*, despite the fact that its holotype, AMNH 26582, is a partial skull preserving parts of the nasal and frontal surface. From published photos of AMNH 26582 [e.g., 2:fig 131] and our own (Fig 6), a flat dorsal surface of the nasals and frontals shows no sign of an attachment scar for an ossicone above or behind the orbit, and presumably it represents a female, i.e., *Q. tungurensis* being sexually dimorphic [this being the case for most extinct giraffids; see 3]. Therefore, the exact position of the ossicone on the skull of *Q. tungurensis* is currently unknown.

Despite its upright ossicone in lateral view, IVPP V 33851 has a prominent curvature in anterior and posterior views, presumably curving inward (medially) (Fig 4). We assumed it to be inwardly curving because most giraffid ossicones either bend medially or have a straight shaft.

The surface texture of IVPP V 33851 suggests extensive coverage of integument. Ossicones of modern giraffes and okapis are covered by integument and vein impressions along ossicone surfaces [3]. The bony surface of IVPP V 33851 is covered by numerous small pits, as well as an occasional vein impression.

The apex of IVPP V 33851 is rounded and slightly expanded relative to the shaft immediately below, very similar to those of *Honanotherium*. Several large pits as well as numerous smaller pits are present on the apex, indicating highly vascularized integument covering. The lack a smooth or polished surface suggests that at this stage of the growth for IVPP V 33851, the tip of the ossicone was not exposed as bare bones. Immediately below the expanded apex, the ossicone shaft is slightly constricted with a relatively smoother surface texture. This distinctly different textural surfaces between the rounded apex and smooth neck suggest a more active secondary growth at the tip and great degree of vascularization.

The main shaft has a mostly rounded cross-section toward the top but becomes progressively flattened toward the base starting at mid-way point of the shaft, as the anterior and posterior ridges become progressively more prominent toward the base of the ossicone (Fig 5). This flattening of the anterior and posterior edge is especially prominent toward the outwardly curved surface, whereas the medial side maintains its rounded cross section (lower two cross sections in Fig 5).

The most distinct feature of the ossicone in *Qilin tungurensis* is a prominently enlarged flange at the base (Figs 3–5). We are unable to ascertain if this flange is anteriorly or posteriorly positioned because of the perfectly upright orientation of the main shaft. Several giraffids are known to have both anterior and posterior ridges on the ossicones [15,33]. Solounias [3:261] also remarked that the “ossicone base more commonly has large bumps (excrescences) posteriorly or anteriorly (*Giraffokeryx* or *Sivatherium*, respectively).” The bumps are present on both anterior and posterior faces of the ossicone in IVPP V 33851, and therefore they do not allow a clear decision in its orientation either. We tentatively regard the extensive overgrowth as anterior, pending further evidence. We also note that on *Giraffokeryx*, a more extensive posterior overgrowth is seen as if to strengthen the buttress of the ossicone attachment to the skull, although a somewhat less anterior overgrowth is also seen at the base.

The question of whether or not this overgrowth represents a separate ossification center is difficult to answer. The base of the ossicone shows a homogeneous surface for the attachment to the skull. The surface texture of the Tunggur specimen shows no sign of sutures between the overgrowth (anterior flange) and main shaft. It thus seems prudent to assume a single (one pair) ossicone for IVPP V 33851 despite the fact that this overgrowth is separate from the main shaft by a distinct hump, as if representing a separate ossification center (i.e., four ossicones). Presence of small bumps on the posterior edge is also similar to those seen in *Decennatherium* [14,34].

However, a pair of separate, small ossification centers in front of the main ossicones are seen in *Decennatherium* [34], *Schansitherium*, and *Birgerbohlinia*, or behind the main ossicone in *Bramatherium*. In the case of *Schansitherium*, the anterior ossicones are so close to the base of the main ossicones that the anterior ossicones are fully fused to the main ossicones at the base and thus has the appearance of an anterior branch of the main ossicone, without any sign of a suture zone between the anterior and posterior ossicones, although in younger individuals the sutures are readily visible.

We note that when a second pair of ossicones are present, either anterior or posterior to the main ossicone pair, they have a rounded or oval cross section and shaped like a cone, as seen in *Schansitherium* and *Birgerbohlinia*. This is in contrast to IVPP V 33851, whose flat, plate-like flange is more like an extension of anterior ridge of the main ossicone as opposed to a discrete ossification in *Schansitherium* and *Birgerbohlinia*. We thus favor an interpretation of overgrowth of the main shaft for the anterior flange of IVPP V 33851, i.e., only one pair of main ossicones are present without a separate small, secondary anterior ossicone pair.

Opposite to the anterior flange discussed above, there is also a posterior but much weaker ridge. This posterior ridge is undoubtedly an overgrowth, rather than being a separate ossification center. Although missing much of its basal part along the posterior ridge, its original size and shape is likely much less extensive than the anterior one because of its thin-bladed basal spur.

Along much of the anterior surface of the flange and the posterior ridge, there are knobby, bulging growth areas, especially toward the base of the ossicone [epikouron of 21]. Such bulges have also been documented in *Honanotherium* [21]. Solounias [3:261] remarked that with the possible exception of *Bramatherium*, ossicones appear to be not branched and the apparent branches in sivatheres are large bumps (excrescences) occurring in regular positions. Therefore, when more than one pair of the main ossicones occur, they are due to separate ossification centers located away from the main ossicone, as in *Giraffokeryx* and *Schansitherium*. In the case of *Schansitherium*, it is clearly two fused ossicones, rather than a single branched one, on each side of the skull. If this is the case, the anterior overgrowth in IVPP V 33851 may be similar to the cases of large bumps, which occur at anterior and posterior bases in the main ossicones of *Giraffokeryx* and *Sivatherium*.

The ventral surface of the ossicone in IVPP V 33851 has highly vascularized, irregular growth texture of trabecular bones, presumably due to extensive cartilaginous contact with the frontal bone that is not fused to ossicone (Figs 3C and 7). As such, it indicates that IVPP V 33851 is a juvenile individual. The ventral surface has an irregular, concave surface that corresponds to a domed frontal bone at the frontal-ossicone contact zone. At the deepest (top) part of this concave surface, immediately below the main shaft, is a large opening about 6 mm in maximum diameter (Fig 3C). This opening penetrates deeply into the main shaft of the ossicone and forms a long, central tube reaching to about 25% of the total shaft length (Fig 7). Small, short side branches are also visible in the CT x-ray images, suggesting functions of a vasculature. It is not clear if this tube is connected to a frontal sinus (if present). In overall morphology, IVPP V 33851 is similar to the undersurface of the giraffe and okapi ossicones. Note that small frontal sinuses occur in all giraffids. These are at the base of the ossicone. This is a morphology entirely different from what is observed in *Baramtherium, Okapia* and *Giraffa* where the sinuses are very large.

**Remarks**: In his original description of *Palaeotragus tungurensis*, Colbert [1:4] noted “tangible differences” in the premolars between his new Inner Mongolian species and the rest of then known species of *Palaeotragus*. In particular, he was impressed by the “extraordinarily well-developed anterior and posterior stylar folds” (his “premolar folds”) as a defining feature of his new species. However, he was also mindful of considerable variations in this character among his Tunggur specimens, and as such, was unsure how much weight he should place on this character.

Some of the above variations may be due to the existence of more than one taxon at Wolf Camp. Of the 12 specimens (AMNH 26582–26593) from Wolf Camp originally listed under *Palaeotragus tungurensis* by Colbert [1], Hou [2] singled out three specimens (AMNH 26589, right dentary with dp2–4 and m1; AMNH 26592, right dentary with p2-m3; and AMNH 26593, left dentary with m1-3) that she considered similar to *Palaeotragus* cf. *P. coelophrys*, a common taxon in northern China. She thought these three specimens possibly represented a large form similar to her *P.* cf. *P. coelophrys*, but with wider and more robust teeth (also reflected in the robustness of their mandibles; personal observation by NS). Despite the fact that Hou did not explicitly include the above three AMNH specimens in the hypodigm of her *P.* cf. *P. coelophrys*, she clearly recognized the mixed nature of Colbert’s [1] original *Palaeotragus tungurensis*. Additionally, there may even be another ruminant in the AMNH Wolf Camp collection as suggested by astragalar differences (personal observations by NS).

We further note that *Palaeotragus* ossicones typically have a smooth surface, rounded cross section, and sharp apex, which are in sharp contrast to the new Tunggur ossicone. Therefore, despite the uncertainty of the assignment of the new ossicone due to a lack of associated ossicones and dental materials, we consider it more likely that the ossicone belongs to *Qilin* rather than *Palaeotragus*.

From the late Miocene localities of Fort Ternan in Kenya, Churcher [35] recognized a new species, *Palaeotragus primaevus*. Based on its p2-3 morphology, he considers his new species closest to “*P.*” *tungurensis*. Hamilton [4] went further and considered this African species to be a junior synonym of “*P.*” *tungurensis* from Tunggur. However, based on Churcher’s illustration of the upper premolars, “*P.*” *primaevus* seems to lack an inwardly bent parastyle and metastyle seen in “*P.*” *tungurensis*. Harris et al. [36] referred the Kenyan species to *Giraffokeryx*, but Parizad et al. [15] seem inclined to leave the question open whether or not “*P.*” *primaevus* represents a primitive species related to “*P.*” *tungurensis*.

The unique morphology of the new Tunggur ossicone forced us to confront the question of whether or not it is a two-ossicone form or a four-ossicone form. A large anterior flange in IVPP V 33851 shows a distinct hump with its tip being upright and separated from the main ossicone shaft by a saddle. Such a morphology reminds one about the four-ossicone *Schansitherium* with closely spaced anterior and posterior (main) ossicones [2,37].

The anterior and posterior ossicones in *Schansitherium* are so close to each other that when the two are sufficiently enlarged, the bases of the ossicones are fused together. One can therefore envision the possibility of two ossification centers fused into one for IVPP V 33851. Such a scenario may even be likely if the small secondary ossicone is poorly developed relative to the main ossicone (as it is a young individual). Furthermore, in the history of giraffid evolution, four-ossicone taxa are not uncommon, such as various species of *Giraffokeryx*, *Bramatherium*, *Schansitherium*, *Sivatherium, Samotherium*, and *Decennatherium*. Hou et al. [38] remarked that only sivatheres and *Giraffokeryx* have an expansion of flanges at the base of their ossicones.

However, we interpret IVPP V 33851 to be a single pair of ossicones with a large anterior overgrowth partly because of its dental similarity to *Bohlinia*, in addition to its overgrowth morphology. If our association of the dental materials with the ossicone is correct, then a Bohlinini relationship is warranted. Known bohlinins, such as *Bohlinia* and *Honanotherium*, have two ossicones (a single pair). We therefore treat the anterior flange of IVPP V 33851 as a basal overgrowth in a two-ossicone (one pair) condition, not a separate ossification center.

## Phylogenetic discussions

Any coherent phylogenetic framework of the giraffoids must be capable of accounting for four morphological systems: cranial and dental characters, ossicone morphology, limb elongation, and neck elongation (astragalar morphology is also promising but it has not been fully integrated into existing evolutionary frameworks). In principle, these four systems are assumed to evolve independently. Elongation of the limbs, however, may require the elongation of the neck because of the necessity of lowering the head to drink, but the reverse is not necessarily true [3].

As is often the case in phylogenetic studies, no single phylogeny for the giraffoids can account in perfect concordance for all of the character systems, presumably because of the inevitable homoplasies. Therefore, some kinds of parsimony algorithms are typically employed to arrive at a phylogenetic hypothesis that reconcile all of the characters. Published cladistic analyses in the last 50 years vary considerably in their selections of characters and sampling of taxa, and as a result, no consensus has emerged [3,4,14,16,17]. It is beyond the scope of this paper to propose a comprehensive phylogeny, and we will only make some comments related to the subfamily Giraffinae. In the follow discussions, we will comment on the above four morphological systems within the subfamily Giraffinae to the extent they are known for our Inner Mongolian taxon.

### Dental characters

**Fig 8:**
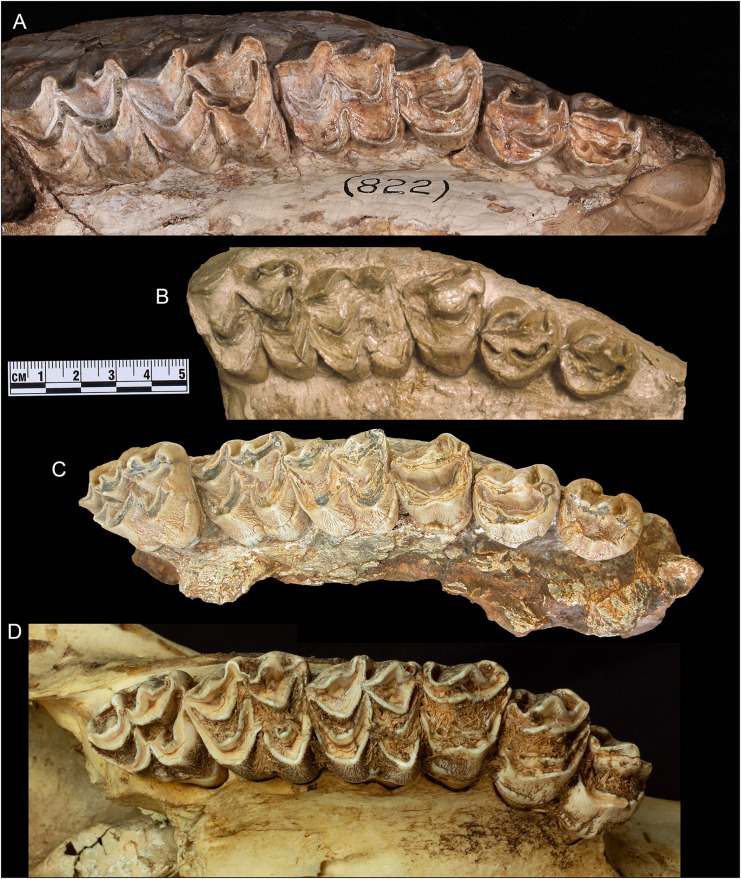
Comparison of upper cheek teeth of *Qilin tungurensis* [1] (A, B), *Bohlinia attica* [39] (C), and *Giraffa* (D). (A) *Q. tungurensis*, AMNH 26582, right P2-M3, holotype from Wolf Camp; (B) *Q. tungurensis*, AMNH 26586, partial palate with right P2-M2, paratype from Wolf Camp; (C) *B. attica*, SNSB-BSPG AS II 640, right P2-M3 (reversed from right side), holotype; (D) *Giraffa camelopardalis*, AMNH Mammalogy uncatalogued (reversed from left side). Scale is for A–C and D has no scale. Photos by Wei Gao (A) and Nikos Solounias (B, C, D).

In his original description of *Palaeotragus tungurensis*, Colbert [1] ascribed his specimens to the genus *Palaeotragus* largely based on his assessment of the dental characters. Although he noted its distinct P2-3 para- and metastyles (Fig 8A and, 8B), he placed more emphasis on the p3-4 morphology [1:fig 7], which pointed to close similarities to forms such as *Giraffokeryx*, *Samotherium*, and *Palaeotragus rouenii*. Colbert, however, was apparently cognizant that these resemblances in the lower premolars may represent primitive characters. If they are shown to be shared primitive characters, then the p3-4 morphology cannot be used as a basis for grouping with other species.

In a first cladistic analysis of giraffoids, Hamilton [4:206] noted that *Qilin tungurensis* agrees dentally with *Giraffokeryx punjabiensis* because of their forked p4 central labial cusp plus other features, as also occurring in *Triceromeryx* and *Canthumeryx*. However, he emphasized characters associated with limb and neck lengthening and ossicone morphology over dental morphology, thus placing *Q. tungurensis* at the base of a *Honanotherium-Bohlinia-Giraffa* clade. Solounias [3] highlighted strongly inwardly folded premolar parastyles and metastyles in *Q. tungurensis* that are shared with *Bohlinia attica*, as also noted by Hou [2].

Most recently, the Tunggur specimens have been placed within *Bohlinia* based on having a P2-3 parastyle and metastyle strongly curving in towards the mesostyle, a character also shared with *Bohlinia attica* (Fig 8C) and *Helladotherium duvernoyi* [5]. Even more tantalizing, this character may also be observed in some individuals of living giraffes [40] (Fig 8D). The frequency of this occurrence in modern giraffes is not known, and this character may further support the notion that *Giraffa* evolved from within Bohlinini [3,4]. We note, however, that *Honanotherium* lacks the above curved styles in upper premolars, which may suggest a more basal status in the Bohlinini clade (see also its limb morphology below).

### Ossicone morphology

**Fig 9:**
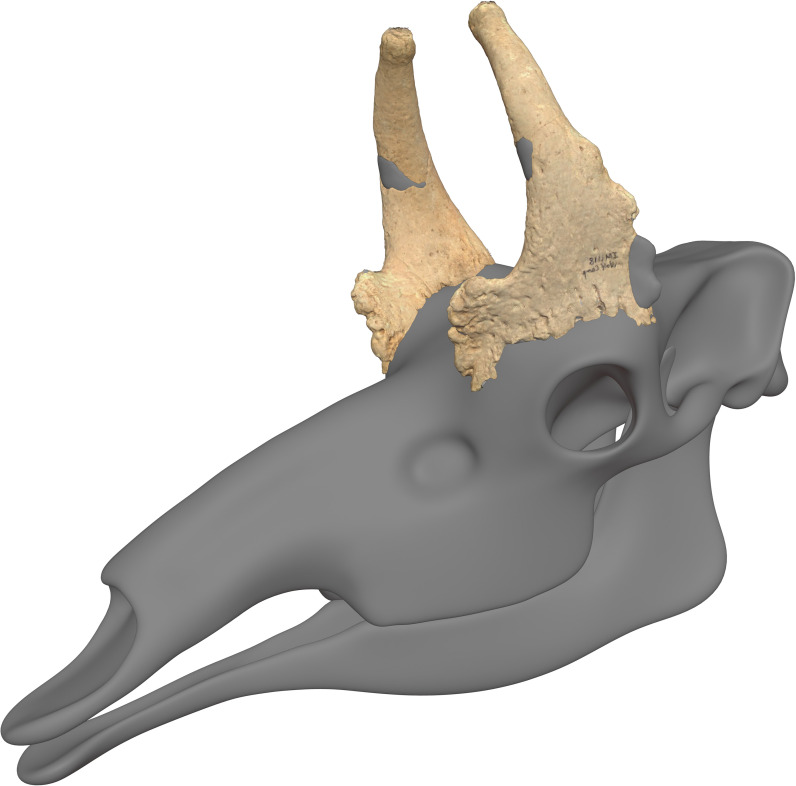
A 3D laser scan model of IVPP V33851 attached to a skull model based on *Bohlinia attica* as described by Parizad et al. [15]. Hand-sculpted skull model (in reduced resolution to serve as a mannequin) and digital manipulation of ossicones (including restoration of missing parts) by Ville Sinkkonen. The models can be downloaded at this site (https://www.morphosource.org/concern/media/000711877?locale=en).

Solounias [3:261] summarized that giraffe ossicones evolved from growth spurs that became detached and formed the small osseous disks seen in juvenile giraffes and okapis. The ossicones then grow rapidly but they do not fuse to the skull until later in life. The frontal grows upward into the growing ossicone, forming a supporting boss, and the base of the ossicone is concave where it contacts the frontal. During the growth, the ossicone mass is full of vasculature and heavily invested integument (see Fig. 7). Later in life, it becomes more compact and often forms a smooth external surface. Our new Tunggur ossicone is thus similar to the growth stage of a juvenile giraffe and okapi. In external morphology, ossicones of living giraffes are characterized by limited epikouron (secondary bone growth) at the tip, posteriorly reclined main shaft, and concave, pitted undersurface where the ossicone attaches to the skull but not fully fused with the frontal [41]. In overall morphology, IVPP V 33851 is remarkably similar to those of modern giraffes as well as to those of Bohlinini (Fig 9).

Our new Tunggur ossicone is also remarkably similar to that of *Bohlinia*. In their descriptions of giraffid materials from Late Miocene Nikiti sites in Macedonia, Greece, Kostopoulos et al. [33] recognized two species of *Bohlinia*, *B. attica* (including NKT-52 and NKT-145) and a new species *B. nikitiae* (holotype NKT-147), both from Nikiti 1 locality. They contrasted the ossicone morphology of these two species [33:fig 13]. Parizad et al. [15], on the other hand, envisioned only one species from these localities in Greece, *B. attica*, which is followed in this study. From Pikermi, Greece, another partial skull of *B. attica* (NRM M4420) was described by Bohlin [11].

The ossicone of *Qilin tungurensis* may also share similarity to *Schansitherium tafeli*, such as forked ossicones and irregularities in the anterior branch. In *Schansitherium*, the irregularities are more confined to formations of small horizontal plates stalked on top of each other. In *Q. tungurensis*, however, the irregularities are more disorganized. In the posterior ossicone branch, these two forms differ in their apex morphology. *Q. tungurensis* has a blunt apex as in *Bohlinia* and *Giraffa*, in contrast to a pointed apex in *Schansitherium*. Finally, *Schansitherium* often have elongated grooves on the surface of the ossicone. These observations suggest that the Tunggur form, depending on how the homology of its anterior flange is interpreted, may be considered as a key taxon and ancestral to at least two distinct lineages of Giraffidae: the Bohlininae and the Samotheriinae. As such it possibly holds an evolutionary key position for the Middle and Late Miocene members of the family.

*Qilin tungurensis* shares with *Giraffa*, *Bohlinia*, and *Honanotherium* the following features: an upright, single pair of ossicone, a rounded tip with a slight constriction below the tip, highly vascular tip surface with secondary growth, and well-developed frontal sinus that penetrate into the shaft of ossicone (this latter feature cannot be observed for lack of a male skull in *Qilin*; *Bohlinia* also appears to lack a frontal sinus). *Q. tungurensis*, however, possesses a unique morphology of its own (autapomorphy for the genus), such as a distinct posterior ridge and a prominent anterior flange, both with multiple bumps. These autapomorphies may suggest an early side branch of the Bohlinini clade in northern China that did not directly lead to the Late Miocene *Bohlinia*.

### Limb lengthening and a deep trough on metacarpal

In his cladogram, Hamilton [4] placed “*Palaeotragus*” *tungurensis* at the base of a clade of *Honanotherium* and *Bohlinia* leading up to *Giraffa*. He listed progressive elongation of the limbs as the main synapomorphy for this clade. Solounias [3:fig 21.7] was able to substantiate Hamilton’s hypothesis by illustrating a composite metacarpal of *Q. tungurensis* (AMNH 92306 and AMNH uncatalogued, field number 808; these specimens were not listed by Colbert, 1936 in his original description of this species), despite its incomplete nature. Gaudry [42] illustrated *Bohlinia attica* Gaudry and Lartet, 1856 from Pikermi, showing lengthened metapodials, which is one of the bases for aligning it with *Giraffa*. Perhaps consistent with its dental characters, *Honanotherium* also has elongated metapodials, although they are shorter and wider than those of living giraffes [11].

Giraffe limb evolution is apparently more complicated than simple elongations [3]. In *Bohlinia attica*, for example, the metacarpal remains long but seem to have broadened in width as well. In addition to limb elongation, a deep posterior trough near the proximal end of the metacarpal further complicate the picture. This deep trough allowed Solounias [3] to further propose the subfamily Bohlininae (=Bohlinini here), as is typified by *B. attica* from Pikermi and *Honanotherium schlosseri*. Presence of this character in *Q. tungurensis*, although not as deep as in *Bohlinia* and *Honanotherium*, is thus suggestive of its membership within Bohlinini. In Giraffini the metapodial trough is reduced, which prompts Solounias [3] to contemplate an alternative hypothesis of *Giraffa* deriving from *Palaeotragus rouenii* mostly based on their p4 morphology. A more recent review of giraffid metapodials is performed by Ríos et al. [43] and a morphometric analysis by Martino et al. [44].

### Neck elongation

Naturally the most impressive feature of the giraffe is its stunningly long neck, in which the cervical vertebrae exceed >50% of total vertebral column length [45]. The earliest records for the extremely long neck is seen in *Bohlinia attica* [18], followed by some transitional forms. Furthermore, Solounias [3] reasoned that an elongate limb also requires an elongate neck in order for the giraffes to lower their head enough to drink water, although spreading their front legs apart helps too. Unfortunately, the neck status of *Qilin tungurensis* is unknown, although if Solounias’ limb and neck relationship is true, then it may reasonably be assumed that the Tunggur form should also have long necks, a possibility to be tested in the future.

### Astragalar morphology

Solounias and Danowitz [46] made extensive comparisons of astragalar morphology of select giraffids, which were found to reinforce established phylogenetic relationships. Such an inquiry is also expanded to the tarsal joints [47]. The astragalus, therefore, has the potential to open up new avenue of pursuit, especially since this bone is one of the most durable and frequently preserved elements in the fossil records. Unpublished data from the AMNH collection by one of us (NS) suggest that there may be as many as three giraffid taxa based on astragalar morphology, which has the potential to enhance our understanding of giraffid evolution.

### Overall considerations

**Fig 10:**
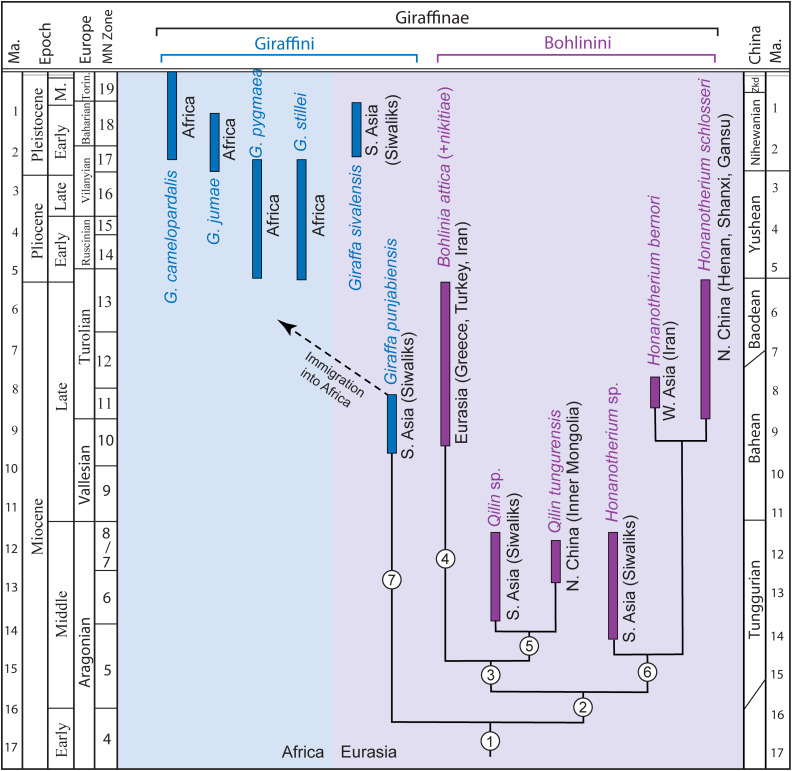
Geographic distributions and chronologic ranges of selected taxa of Giraffinae. Our estimated relationship and potential shared derived characters for Bohlinini follow those by Hamilton [4], Solounias [3], and Solounias and Danowitz [5]. Taxonomy and chronology of giraffes from northern Eurasia follow Parizad et al. [15], Hou [2], Solounias and Danowitz [21], and this paper, Indian Subcontinent follow Solounias and Danowitz [5], and those from Africa follow Harris et al. [36]. Potential shared derived character may include: **node 1** (Giraffinae), limbs very long and slender, necks long, upright single pair of short ossicone terminating in a knob and constriction below knob, highly vascular tip surface with secondary growth; **node 2** (Bohlinini), modestly deep trough on metacarpal; **node 3**, P2-3 para- and metastyles prominent and bending toward mesostyle; **node 4**, increased body size; **node 5**, frontal sinus penetrating into ossicone shaft, ossicone with prominent anterior flange, anterior and posterior bumps at base; **node 6**, increasing body size plus additional characters in different species; **node 7** (Giraffini), cervical 7 with ventral tubercle, extreme elongation of limbs and necks, secondary reduction of trough on metacarpal, increase of body sizes in later species (characters for living *Giraffa*; conditions for early species of *Giraffa* are less clear).

Despite the fact that no articulated skeleton exists for *Qilin tungurensis*, there is enough evidence to allow a firm membership assignment within Bohlinini, assuming the isolated elements mentioned above belong to the same taxon. Of the above four morphological systems, three (teeth, ossicone, and limbs) are consistent with a placement within Bohlinini for *Q. tungurensis*. If our assessments above are correct, it is perhaps no coincidence that *Qilin* falls within the basal Giraffinae (Fig 10).

Bohlin [11] has long recognized a close relationship of his *Honanotherium* with *Orasius* (later named *Bohlinia*), and together with living *Giraffa*, these have traditionally constituted the subfamily Giraffinae. In his first cladogram, Hamilton [4] recognized three basal giraffines: “*Palaeotragus*” *tungurensis*, *Honanotherium*, and *Bohlinia* in a successive phylogenetic arrangement advancing toward *Giraffa*. Successive characters in support of such an arrangement include: very long and slender limb, posterior position of ossicones, lengthening of the neck, well-developed frontal sinus, plus others.

In his new giraffid phylogenetic framework, Solounias [3:fig 21.1], while proposing the distinct subfamily Bohlininae, suggested two alternatives for the evolution of modern *Giraffa*: 1, a traditional relationship of *Bohlinia* and *Giraffa* (node 12, “*Bohlinia attica* [*Giraffa camelopardalis*]”) and 2, a discrete subfamily Giraffinae at the terminal end of the cladogram closely related to *Palaeotragus rouenii* (node 29). Solounias’ proposal was strongly influenced by his evaluation of the metapodial morphology. However, his analysis was not subject to modern phylogenetic analysis (such as parsimony algorithms). Solounias’ novel scenario of separating the giraffines from the bohlinines does not gain traction, and recent cladistic analyses that do balance all morphological characters tend to favor the traditional view of a Giraffini-Bohlinini sister relationship [4,14,18] or something close to it [17].

Solounias [3] recognized that while the traditional relationship of bohlinines + giraffines has the advantage of accounting the extremely long neck and metapodials as evolving once, one has to accept a reversal in reduction of the deep metacarpal trough to almost none in modern giraffes. Solounias [3] also suggested that the strongly curved-in styles of the upper premolars seen in bohlinines must have been lost in *Giraffa* later, although individuals of living giraffe in the AMNH collection (Fig 8D) and some published records [40] seem to show such character. A broader survey of this character is needed to ascertain its variation. Although such an evaluation for all extinct taxa is still lacking, those by Ríos et al. [14,17] were first attempts at evaluations of select taxa and characters. Their parsimony analyses do seem to bear out the above traditional view.

**Fig 11:**
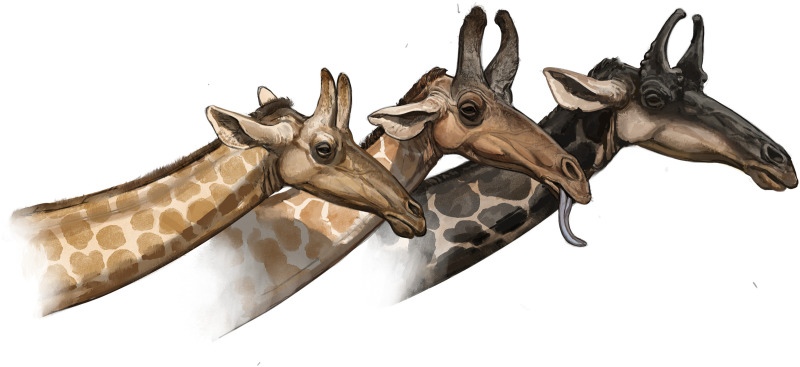
Artist reconstructions of *Qilin tungurensis*: An adult male (right) and younger males (middle and left) with less developed ossicones. Note females did not have ossicones in the early-middle Miocene. Art by Ville Sinkkonen.

Our new ossicone described herein is thus of importance in providing fresh insights (Fig 11). All evidence seems to strongly suggest a Bohlinini membership. If the Bohlinini really represents early members the subfamily Giraffinae (tribes Bohlinini + Giraffini), then the new *Qilin* from Tunggur may belong to one of the first records of the subfamily in the latest Middle Miocene because all other known records of the Bohlinini occur in the Late Miocene (Fig 10), except two undescribed taxa from the Siwaliks of Pakistan [5]. Inner Mongolia in northern China is thus of unique zoogeographic significance in the origin of Giraffinae.

## Conclusions

The new fossil ossicone from the Wolf Camp area in the Middle Miocene Tunggur Formation of Inner Mongolia, northern China, can be referred to a formerly named species *Palaeotragus tungurensis* Colbert, 1936, previously described from cranial, dental, and postcranial materials of the same fossil site. An almost perfectly preserved ossicone presents novel morphology unlike any known so far and a new generic name, *Qilin*, is warranted, as it has long been suspected by previous workers. *Qilin tungurensis* adds important evidence that this Middle Miocene record from Inner Mongolia represents a key taxon in the evolution of subfamily Giraffinae, which includes two tribes, Giraffini and Bohlinini, and the living giraffes, *Giraffa*, falls in the former. Dental morphology of *Q. tungurensis* strongly supports its membership within Bohlinini with a unique shared-derived character of P2-3 para- and metastyles bending inward to mesostyle. Limb morphology of *Q. tungurensis* also strongly suggests membership in Bohlinini because of a slender limb and a modestly deep trough on the posterior surface of the metacarpals. Although no neck vertebrae are known for *Q. tungurensis*, its long and slender limbs are consistent with an elongated neck, as is known for other members of Bohlinini, such as *Bohlinia attica*. Combined with knowledge about its dental and limb morphology, the new Wolf Camp ossicone indicates an important stage of basal giraffine evolution, and contributes to a better understanding of its chronology and zoogeography.
